# Intravenous Hydration and Associated Outcomes in Patients With Sickle Cell Disease Admitted With Vaso-Occlusive Crises: A Systematic Review

**DOI:** 10.7759/cureus.54463

**Published:** 2024-02-19

**Authors:** Sagar Pandey, Ernestine Faye S Tan, Amulya Bellamkonda, Binit Aryal, Sailesh Karki, Gouthami Boddu, Ranjit Sapkota, Madhav Changela, Madhumati Kalavar

**Affiliations:** 1 Internal Medicine, One Brooklyn Health System/Interfaith Medical Center, Brooklyn, USA; 2 Hematology and Oncology, One Brooklyn Health System/Interfaith Medical Center, Brooklyn, USA

**Keywords:** sickle cell disease (scd), prolonged length of hospital stay, review article, fluid overload, vaso-occlusive crisis, adverse event, intravenous fluids, sickle cell crisis

## Abstract

Acute painful vaso-occlusive crisis (VOC) is the common presentation of sickle cell disease (SCD) leading to emergency room visits, admissions, morbidity, mortality, and negative impacts on quality of life. Among various treatment approaches commonly employed to manage the condition, intravenous (IV) hydration is also frequently used in emergency and inpatient settings. Although helpful to overcome dehydration, IV hydration often leads to adverse outcomes like fluid overload, pulmonary edema, increased length of stay, transfer to intensive care unit, new oxygen requirement, etc. Small-scale retrospective studies are conducted to study the outcomes of IV hydration but have failed to conclusively demonstrate its benefits as well as choice of IV fluids, rate of IV fluid replacement, etc. We conduct this review as an attempt to summarize the available evidence on the role and utility of IV hydration in sickle cell crises along with reported adverse outcomes.

## Introduction and background

Sickle cell disease (SCD) is a group of inherited blood disorders that affects the hemoglobin protein inside red blood cells (RBCs) responsible for transporting oxygen. It results from a point mutation in the gene encoding the beta-globin chain leading to the substitution of hydrophilic glutamic acid to hydrophobic valine residue at the sixth codon and forming a mutated hemoglobin tetramer known as sickled hemoglobin (HbS) [1,2]. This results in the polymerization of HbS upon tissue deoxygenation forming long fibers increasing cellular rigidity, and distortion of red cell membrane, promoting sickling and premature hemolysis. Furthermore, polymerization of the deoxygenated HbS results in vaso-occlusion and adherence to post-capillary venules leading to ischemia, intracellular energy failure, and dehydration [2,3]. Presentation and severity of SCD vary depending upon the homozygosity of HbS or mixed heterozygosity with other forms of hemoglobin (HbSC disease, HbS-beta thalassemia, etc.) along with the presence of fetal hemoglobin which replaces HbS and interferes with polymerization [1,2]. Hypoxia, infection, fever, acidosis, dehydration, pregnancy, menstruation, obstructive sleep apnea, pain, anxiety, depression, alcohol consumption, and physical exhaustion have been described as precipitators of sickling [4].

SCD is particularly common among descendants from sub-Saharan African ancestry. Approximately 100,000 Americans are estimated to be affected along with SCD occurring in one out of 365 Black or African American births [5]. In a National Emergency Department (ED) sample database in 2006, combined ED and inpatient hospitalization for SCD amounted to a staggering cost of 2.4 billion dollars. When compared to the cost per 100-person visit, SCD had three times the charges of hospitalization associated with ED visits compared to those with congestive heart failure [6].

Various treatment interventions among patients admitted with sickle cell crises include analgesia with acetaminophen, non-steroidal anti-inflammatory drugs (NSAIDs) or opioids, oxygen in hypoxic patients, and hydration. Judicious use of simple blood transfusion and exchange transfusion have been recommended as interventions in specific clinical scenarios [4,7]. Although hydration has been dogmatically considered an essential component of the management of sickle cell crisis, consensus regarding the effectiveness of intravenous fluid (IVF) therapy has not been established. Additionally, potential risks of the intervention including fluid overload, and pulmonary edema may lead to transfer to the intensive care unit (ICU) and increased length of hospital stay [8-11]. We conduct this review as an attempt to summarize the available evidence on the role and utility of intravenous hydration in sickle cell crises along with reported adverse outcomes.

## Review

Methodology

Search Strategy

We conducted a literature search in the PubMed and Google Scholar databases along with the search for relevant articles in the references section of relevant articles. The search strategy was formulated by conducting isolated searches relating to population, intervention, comparator, and outcome measures (PICO model) pertaining to our study topic. We then combined those isolated search strategies using the Boolean operator “AND” and conducted a final search for relevant articles. The search terms included keywords like sickle cell trait, sickle cell anemia, hemoglobin SC disease, and vaso-occlusive crisis (VOC) for the study population. For study intervention, keywords like intravenous infusions, intravenous hydration, and IVFs were used whereas hospitalization, length of stay, adverse event, outcomes, volume overload, etc., were used for study outcome. All of the studies published till January 25, 2024 were used in the study. A complete search strategy is presented in the appendix.

*Inclusion Criteria*

We included any prospective, retrospective, or clinical trials meeting our inclusion criteria in the review. The patient population included sickle cell patients who were admitted either inpatients or in the emergency department with sickle cell VOC. Administration of IVF was a study intervention and patients who received IVF were compared to those who did not receive IVF. Improvement in pain severity, volume overload, length of hospitalization, hospital-acquired infections, transfer to the inpatient unit, acute kidney injury, new oxygen requirement, acute chest syndrome, etc., were the study outcomes.

**Figure 1:**
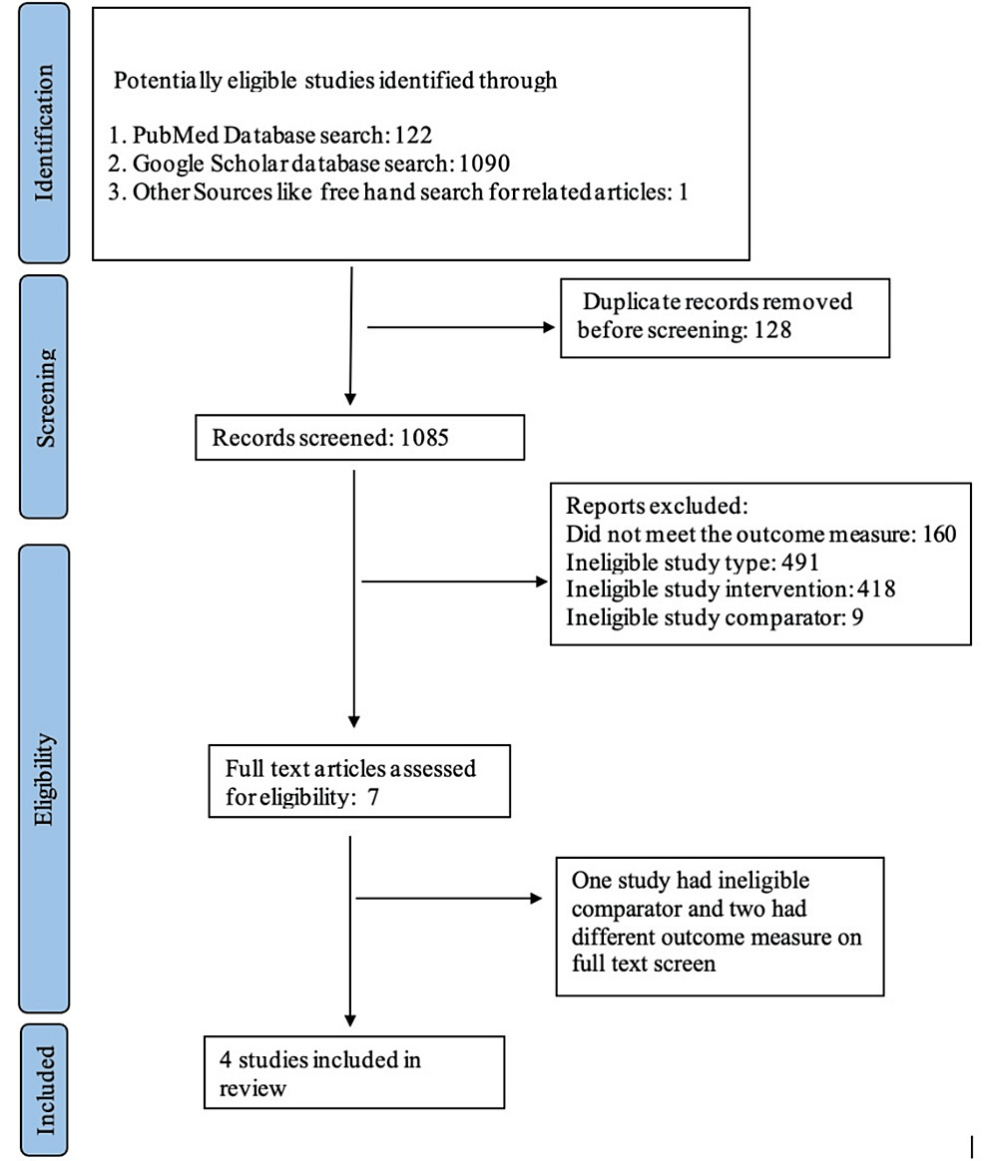
Flow diagram demonstrating selection of studies.

Studies not meeting the specified inclusion criteria were excluded from the study. Study findings were presented using a narrative synthesis approach. A detailed flow diagram depicting the selection of studies is presented in Figure 1.

Data Screening and Extraction

A group of four authors (SP, EFT, AB, BA) was responsible for screening the relevant database for articles meeting the inclusion criteria for the systematic review. The initial search was carried out using the formulated search strategy. Search results were initially screened using title and abstract screening which narrowed down the total number of articles eligible for full text screening. A total of four studies were included in the review after full-text screening. Another group of four authors was responsible for the assessment of the quality of studies included in the review. Any disagreement between the authors regarding study inclusion and quality assessment was resolved after discussion in the group. Zotero, a free and open-source reference management software was used for the data screening and extraction process.

Assessment of Quality of Studies

Newcastle-Ottawa quality assessment tool for cohort studies was used for the assessment of the quality of studies included in the review. Quality of studies was assessed under three key domains namely the selection of study groups, comparability of study groups, and ascertainment of the outcome of interest for the study cohort. Studies were given points in the form of a number of stars after answering the numbered items under each domain. An individual study could be given a maximum of one star for each numbered item within the selection and outcome domain whereas a maximum of two stars could be given for the comparability domain. The scale does not provide a strict cutoff for study quality but studies with higher scores were considered higher quality [12]. Table 1 demonstrates the assessment of the quality of studies included in the review.

**Table 1:** Quality assessment of studies using a Newcastle-Ottawa scale

Study	Selection domain	Comparability domain	Outcome domain	Study quality
Gaartman et al. [8]	3 stars	1 star	3 stars	High
Carden et al. [9]	3 stars	0 star	3 stars	Moderate
Gaut et al. [10]	3 stars	0 star	3 stars	Moderate
Haimed et al. [11]	3 stars	0 stars	3 stars	Moderate

Results

IV Hydration in the Management of Sickle Cell VOC

A tabulated summary of studies exploring the implications of IV hydration in the sickle cell population with VOC is presented in Table 2.

**Table 2:** Summary of studies exploring the role and implications of IV therapy in the sickle cell population with VOC HbSS: sickle cell anemia, HbSC: sickle‐ hemoglobin C disease, HbSβ^0^‐thal: hemoglobin S‐beta^0^ thalassemia, HbSβ^+^‐thal: hemoglobin S‐beta⁺ thalassemia, NS: normal saline, 0.65%NS: 0.65% normal saline, D2.5%: dextrose 2.5%, 1/2NS: half normal saline, LR: ringers lactate, D5NS: dextrose 5% and normal saline, D51/4NS: dextrose 5% and 1/4 normal saline, KCL: potassium chloride, IV: intravenous, ICU: intensive care unit.

Study authors and year of publication	Type of Study	Study Population	Sample Size/ total encounters	Study Setting	IV hydration used	Outcome measures
Gaartman et al. (2021) [8]	Retrospective review	HbSS, HbSC, HbSβ^0^‐thal and HbSβ^+^‐thal	100/230	Inpatient	NS, 0.65% NS, D2.5 + 1/2NS	Fluid overload and risk factors in the development of fluid overload
Carden et al. (2019) [9]	Retrospective Review	HbSS or HbSβ^0^-thal	400/400	Inpatient and pediatric emergency room	NS, LR 1/2NS D5NS D5 1/2NS D5 1/4NS D5NS + KCl 20 mEq, D5 + 1/2NS + KCl 20 mEq	Improvement in pain severity
Gaut et al. (2020) [10]	Retrospective Review	HbSS, HbSC	49/157	Inpatient	Various IV fluids	Complications of sickle cell, including acute chest syndrome, acute kidney injury, new oxygen requirements, ICU transfers and overall risk of adverse events
Haimed et al. (2022) [11]	Retrospective Review	HbSS, HbSC	161/617	Inpatient	Various IV fluids	Length of hospital stay

Volume Overload

Fluid therapy has been considered a cornerstone of management in sickle cell patients during VOC. Poor hydration status can cause increased plasma osmolality leading to RBC dehydration, resulting in increased HbS polymerization and vaso‐occlusion [13]. However, fluid therapy may have its complications, and one that carries potentially significant morbidity is fluid overload.

Gaartman et al. investigated the incidence of fluid overload following IVF therapy for patients with VOC. In this retrospective study, 100 patients admitted for VOC were analyzed over a period of two years. Subjects individually received a total of three liters in 24 hours, for 72 hours, after which hydration was discontinued or tapered down. The primary outcome was the presence of clinical manifestations of fluid overload, such as crackles, shortness of breath, pulmonary or peripheral edema, or the need for diuretic use. In this study, 21% of the study population developed fluid overload. Although underlying diastolic dysfunction in patients with SCD was hypothesized as a reason behind volume overload, no differences in diastolic dysfunction were found in patients with and without fluid overload. Rather, a history of volume overload itself was found to be an independent predictor for recurrence of fluid overload (p = 0.0017). Blood transfusion during admission (one or two units of 300 mL RBCs) was also found to be a significant risk factor for the adverse outcome (p = 0.004). Lastly, an association of volume overload with the amount of IVF administered could not be analyzed as IVF had to be prematurely stopped due to the development of volume overload [8].

Carden et al. in an attempt to determine optimal IVF to increase RBCs deformability in VOC demonstrated that sickled cells exposed to normal saline had increased stiffness, transit times and propensity to microvascular occlusion compared to hypo-osmolar fluids [14]. Furthermore, sickling was favored with a decrease in blood pH and vice versa. Normal saline resulted in hyperchloremic acidosis promoting HbS polymerization and worsening vaso-occlusion [8,15]. However, leading institutions like the American Society of Hematology have not offered a recommendation for or against IV fluids, the route of administration, or the choice of fluids in patients with VOC. Furthermore, it has been stated that the harm may be greater in adults with SCD than in children due to underlying cardiorespiratory compromise [16].

Improvement in Pain Severity

Acute painful VOC is the principal symptom of SCD that causes most of the emergency room visits, admissions, morbidity, mortality, and negative impacts on quality of life [17,18]. Naturally, the main goal of treatment revolves around the relief of the pain. While acute painful episodes are primarily managed with analgesics, the role of IVF in the management of VOC is debated. Carden et al. using a microfluidic device that modeled human microvasculature demonstrated that alteration of extracellular tonicity affected the biomechanical properties of sickled RBCs (sRBCs) thus leading to alteration in its deformability, transit time, and the risks of microvascular occlusion. It was concluded that compared to hyper and hypotonic IVFs, IVFs with intermediate tonicity reduced the risk of vaso-occlusion in experimental models [14]. The findings of the study in invitro microfluidic models are limited by the lack of translative evidence in real-world clinical scenarios in the form of well-structured clinical trials.

A study done by Carden et al. in 2017 studied the effects of hydration on improvement in pain scores among patients presenting with VOC on admission. A total of 400 sickle cell patients with VOC were divided into two groups, those who received fluid bolus in ED and those who did not. Significantly less improvement in pain scores was reported in patients who received normal saline boluses compared to those who did not (p = 0.03). Furthermore, higher rates of inpatient admission (71% vs. 59%; p = 0.01) and longer time spent in ED (5.2 vs 4.8 hours, p = 0.01) were observed in the normal saline bolus group [9].

Length of Hospital Stay

Gaartman et al. emphasized that patients who developed fluid overload had significantly longer hospital stays than those who did not (six days vs. four days; p = 0.037) [8]. Haimed et al. investigated this particular outcome measure in their retrospective study involving 161 patients and 617 hospitalizations over the course of five years. The average hourly IVF rate is compared to their estimated weight-based maintenance IVF (mIVF) rate. The mean IVF volume administered during the total admission and over the first three days of admission were 49.9 mL/kg/day (SD 20.3) and 139.6 mL/kg/day (SD 57.8), respectively. It was found that during the first 3 days of admission, hospital length of stay increased by 0.53 days for each additional 0.5 times the mIVF rate (p < .001; 95% confidence interval (CI): 0.609-0.989), suggesting that excessive fluid administration during a VOE in SCD patients is associated with a prolonged hospital stay. Normal saline with 5% dextrose was used as maintenance fluid in the majority of patients in the study [11].

Other Complications

Due to the paucity of literature regarding a consensus for standardized fluid administration among patients with sickle cell VOC, renal, cardiac, and pulmonary complications are reported with the use of IVF [19]. A study by Gaut et al. explored these outcomes in a retrospective analysis of 157 patient encounters from 49 patients with sickle cell admitted for VOC over the course of four years. The mean total amount of IV fluids administered during hospitalization was 7.4 liters (SD= 9.6), while the mean total amount of fluids, including blood transfusions and oral fluids was 14.2 L (SD= 18.2). The study found a significant association between the development of adverse events like new oxygen requirement, acute chest syndrome, aspiration event, hospital-acquired infection, acute kidney injury, and ICU transfer with the amount of fluid administered. Adverse events correlated with the amount of IVF administered in the first 24 hours, the total amount of IVF administered, and the total amount of fluid intake, including oral fluids and blood transfusions. Other associations were found with dialysis dependence prior to admission and admission to an inpatient service versus an emergency room or observation unit [10]. Similarly, Graham et al. in a review of 21 autopsy cases of sudden/unexpected death among sickle cell patients, found that 71.4% showed significant pulmonary pathology which included pulmonary edema (47.6%), pulmonary hypertension (33.3%) along with right ventricular hypertrophy in one-third of patients [20].

This correlation between high fluid volumes and adverse events during hospitalization emphasizes the risks of aggressive IVF therapy. Furthermore, the underlying compromised renal and cardiopulmonary function in patients with SCD further adds challenge to the body’s compensatory response to fluid challenge [21,22].

To summarize, there remains an evidence gap between maintaining a therapeutic dosage of hydration and avoiding the toxic window, choice of IVF along with rate of IVF administration among patients with SCD with VOC. A novel approach by Carden and associates suggests the use of physiologically balanced salt solutions as IVF, but the lack of controlled trials for its effectiveness in adults limits its use [9]. Lastly, although a weight-based approach to hydration has been investigated extensively in other medical conditions such as sepsis, burns, and hyperglycemic emergencies, a similar guide for sickle cell patients has not been established, and it remains a topic to be explored.

Research Gap on the Role of IV Hydration in Sickle Cell VOC

Although isolated retrospective studies have been conducted to assess the role of intravenous hydration in patients admitted with sickle cell VOC, large-scale clinical trials addressing the clinic question have not been done yet. Due to limitations in sample size, and variable clinical settings with a broad range of study population, the outcomes from these observational studies cannot be reliably extrapolated for managing patients in day-to-day clinical settings. American Society of Hematology guidelines for managing SCD 2020 similarly, have chosen not to provide any recommendations for or against IVF replacement in patients with sickle cell VOC [16]. To date, only available data on the role of IV hydration in SCD patients is in the form of a single randomized clinical trial which was conducted among patients aged 4 to 21 years presenting with sickle cell vaso-occlusive episode in ED. However, the question addressed in the trial was the role of warmed vs non-warmed normal saline on hospital admission rates, pain scores, disposition times, the dosage of opioids, and comfort. Pain score reduction, hospital length of stay, opiate doses, and hospital admission rates were found to be comparable whereas global comfort level was found to be higher among those who received warmed saline (4 vs 3, p-value=0.01) [23]. It is therefore imperative to conduct large-scale clinical trials to assess the role of intravenous hydration among patients with SCD presenting with VOC, not only to formulate universal treatment guidelines but also to prevent the unrestrained use of IV hydration and associated adverse outcomes.

## Conclusions

There remains an evidence gap regarding the standardized choice of therapy to maintain hydration in a therapeutic window along with the impact of IVF on clinical outcomes. Available data on the safety and efficacy of IVF is in the form of small-scale studies along with in vitro experimental models. Large scale well-structured clinical trials to translate the findings of small-scale studies are a must to guide the hydration approach in sickle cell VOC. Clinicians should use clinical judgment for a safe and therapeutic mode of fluid administration by assessing the volume status and cardiorespiratory/renal function of the patient with sickle cell VOC. Lastly, institutional standardized fluid administration protocols in sickle cell VOC would also contribute to guiding safe and effective hydration and minimizing adverse outcomes.

## Appendices

Search strategy

#1. Population:  "Sickle Cell Trait"[Mesh] AND "Anemia, Sickle Cell"[Mesh] AND  "Hemoglobin SC Disease"[Mesh] OR “sickle cell”[tiab] OR “sickle cell crisis”[tiab] OR “vaso-occlusive crisis”[tiab] OR “vaso-occlusive episode”[tiab]

#2. Interventions: "Infusions, Intravenous"[Mesh] OR “hydration”[tiab] OR “fluid*”[tiab]

#3. Outcome: “hospitalization”[tiab] OR “length of stay”[tiab] OR “adverse event*”[tiab] OR “outcome*”[tiab] OR “volume overload”[tiab]

Final search: #1 AND #2 AND #3 

## References

[1] Malowany JI, Butany J (2012). Pathology of sickle cell disease. Semin Diagn Pathol.

[2] Sundd P, Gladwin MT, Novelli EM (2019). Pathophysiology of sickle cell disease. Annu Rev Pathol.

[3] Ilesanmi OO (2010). Pathological basis of symptoms and crises in sickle cell disorder: implications for counseling and psychotherapy. Hematol Rep.

[4] Ashorobi D, Ramsey A, Yarrarapu SNS, Bhatt R (2024). Sickle Cell Trait. https://www.ncbi.nlm.nih.gov/books/NBK537130/.

[5] (2024). Data & statistics on sickle cell disease. Control Prev.

[6] Lanzkron S, Carroll CP, Haywood C Jr (2010). The burden of emergency department use for sickle-cell disease: an analysis of the national emergency department sample database. Am J Hematol.

[7] Novelli EM, Gladwin MT (2016). Crises in sickle cell disease. Chest.

[8] Gaartman AE, Sayedi AK, Gerritsma JJ (2021). Fluid overload due to intravenous fluid therapy for vaso-occlusive crisis in sickle cell disease: incidence and risk factors. Br J Haematol.

[9] Carden MA, Brousseau DC, Ahmad FA (2019). Normal saline bolus use in pediatric emergency departments is associated with poorer pain control in children with sickle cell anemia and vaso-occlusive pain. Am J Hematol.

[10] Gaut D, Jones J, Chen C, Ghafouri S, Leng M, Quinn R (2020). Outcomes related to intravenous fluid administration in sickle cell patients during vaso-occlusive crisis. Ann Hematol.

[11] Haimed A, Weiss R, Kwon S, Bhat R, Badawy SM (2024). Intravenous fluid therapy and hospital outcomes for vaso-occlusive episodes in children, adolescents, and young adults with sickle cell disease. Pediatr Blood Cancer.

[12] (2024). The Newcastle-Ottawa Scale (NOS) for assessing the quality of nonrandomised studies in meta-analyses. https://www.ohri.ca/programs/clinical_epidemiology/oxford.asp.

[13] Brugnara C (1995). Erythrocyte dehydration in pathophysiology and treatment of sickle cell disease. Curr Opin Hematol.

[14] Carden MA, Fay ME, Lu X (2017). Extracellular fluid tonicity impacts sickle red blood cell deformability and adhesion. Blood.

[15] Bookchin RM, Balazs T, Landau LC (1976). Determinants of red cell sickling. Effects of varying pH and of increasing intracellular hemoglobin concentration by osmotic shrinkage. J Lab Clin Med.

[16] Brandow AM, Carroll CP, Creary S (2020). American Society of Hematology 2020 guidelines for sickle cell disease: management of acute and chronic pain. Blood Adv.

[17] Platt OS, Thorington BD, Brambilla DJ, Milner PF, Rosse WF, Vichinsky E, Kinney TR (1991). Pain in sickle cell disease. Rates and risk factors. N Engl J Med.

[18] Steinberg MH (2006). Pathophysiologically based drug treatment of sickle cell disease. Trends Pharmacol Sci.

[19] Ojo AS, Ojukwu S, Asmare W, Odipe O, Larbi D (2022). Intravenous fluid administration and the risk of adverse outcomes in sickle cell disease patients hospitalized for vaso-occlusive crisis. J Hematol.

[20] Graham JK, Mosunjac M, Hanzlick RL, Mosunjac M (2007). Sickle cell lung disease and sudden death: a retrospective/prospective study of 21 autopsy cases and literature review. Am J Forensic Med Pathol.

[21] Sharpe CC, Thein SL (2014). How I treat renal complications in sickle cell disease. Blood.

[22] Gladwin MT, Sachdev V (2012). Cardiovascular abnormalities in sickle cell disease. J Am Coll Cardiol.

[23] Quarrie RP, Stoner MJ, Choueiki JM, Bonsu BK, Cohen DM (2020). Clinical impact of warmed intravenous saline in sickle cell patients with vasoocclusive episodes. Pediatr Emerg Care.

